# Self-efficacy, Knowledge and Outcomes Expectations Foot Self-care among Diabetic Patients with High-Risk Feet in Jordan

**DOI:** 10.2174/0115733998328984240923115409

**Published:** 2024-11-29

**Authors:** Yahya Najjar, Kathleen Finlayson, Qusai Aljarrah, Sameh Moosa, Mohammed ALBashtawy, Osama Alkouri, Anas Ababneh

**Affiliations:** 1 Zarqa University College, Al-Balqa Applied University , Zarqa, Jordan;; 2 Centre for Healthcare Transformation, Queensland University of Technology, Brisbane, Australia;; 3 Department of General and Vascular Surgery, King Abdullah University Hospital (KAUH), Jordan University of Science and Technology (JUST), Irbid, Jordan;; 4 National Center for Diabetes, Endocrinology, and Genetics, Amman, Jordan;; 5 Department of Community and Mental Health, Princess Salma Faculty of Nursing, Al al-Bayt University, Mafraq, Jordan;; 6 Faculty of Nursing, Yarmouk University, Irbid, Zip Code: 21163, Jordan;; 7 Applied Science Research Center, Applied Science Private University, Amman, Jordan

**Keywords:** Diabetic foot ulcers, foot care confidence, foot care outcomes expectations, self-efficacy, diabetes mellitus, glycated hemoglobin

## Abstract

**Introduction:**

Patients with high-risk feet and diabetes are prone to develop diabetes-related foot ulcers (DFUs). To prevent DFUs and their costs and impacts on diabetes mellitus (DM) patients’ productivity and quality of life, patients’ foot self-care (FSC) practices are essential to screen for DFUs. Our study aimed to assess the self-efficacy and outcome expectations about FSC and evaluate the knowledge about DFUs among patients with DM in Jordan.

**Methods:**

A cross-sectional study was conducted with DM patients who attended two diabetes clinical centers in Jordan in the period from October 2022 to April 2023 in which data were collected through a self-administered questionnaire developed to assess patients’ understanding of DFUs, foot care outcomes expectations (FCOE), and self-efficacy about FSC.

**Results and Discussion:**

The final sample included data from 107 patients. Patient awareness of DFUs and FCOE was good, and the level of self-efficacy for FSC was moderate to high. Multiple linear regression revealed that age was negatively associated with lower self-efficacy for FSC (*β* = -0.22, *p* = 0.03) and both confidence (self-efficacy) in FSC and living arrangements (*i.e.*, living with family) were positively associated with higher FCOE (*β* = 0.19, *p* = 0.04 and *β* = 0.39, *p* <0.001, respectively). Diagnosis of renal failure, diagnosis of retinopathy, elevated levels of glycated hemoglobin, and the settings in which the patient is receiving care for DM were positively associated with an understanding of DFUs. Both age and history of heart attacks and coronary artery disease were negatively associated with realizing DFUs (*β* = -0.26, *p* = 0.007, and *β* = -0.18, *p* = 0.045, respectively).

**Conclusion:**

Patients with diabetes mellitus in Jordan have good FCOE, good awareness of DFUs, and a moderate degree of self-efficacy to execute FSC. Health education and self-efficacy programs should focus on older adults with a history of heart attacks to boost their understanding of DFUs and raise their sense of self-efficacy around FSC.

## 1 INTRODUCTION

Diabetes mellitus (DM) is a metabolic disease necessitating both consistent medical care and education to enhance a patient’s self-care and ability to screen and monitor his/her health condition [1]. DM complications are a significant public health concern with high rates of mortality and morbidity [2]. Among the complications are diabetic foot diseases, recognized as the most important and costly complication [2]. In Arabian countries, 4 -11% of patients with DM have diabetic foot ulcers (DFUs) [3].

Patients’ adherence to foot self-care can reduce DFU morbidity [4]. Appropriate foot care practice among DM patients should be adopted to prevent foot ulcers and subsequent lower limb amputations [2]. Among the most important factors that affect foot self-care (FSC) is patients’ knowledge about DFUs [3]. Furthermore, the quality of FSC can be affected negatively by the lack of adequate knowledge of FSC, which increases the risk of developing diabetic foot (DF) complications [5].

One of the most important contributors to DFUs development is diabetic neuropathy (DN) [6, 7]. To minimize DF complications, especially those leading to amputations, DM patients should be knowledgeable about DM and its complications [8]. Furthermore, a patient’s understanding of the causes of DFUs can foster appropriate FSC [9]. Therefore, assessing the knowledge level of FSC practices and knowledge about DFUs among DM patients is important. Furthermore, self-efficacy is an integral determinant of DM self-care practice [10], as self-efficacy was evident to influence the confidence level to perform FSC and, therefore, enhancing FSC behavior [11].

Screening and early diagnosis of DFUs, in addition to educating DM patients about DFUs, is necessary to reduce the risk of DFUs in those patients [7]. In addition, understanding the causes of DFUs in DM patients can assist in recognizing high-risk feet patients early so that the costs of treating DFUs complications can be reduced, as well as preventing loss of DM patient productivity and enhancing health-related quality of life [12]. To the best of our knowledge, this is the first study conducted in Jordan that aimed to assess the knowledge about FSC and knowledge about DFUs’ development among DM patients as well as assessing the outcomes expectations of DM patients from performing FSC and self-efficacy of undertaking FSC. Furthermore, the study aimed to assess to what extent the demographic and clinical variables of DM patients are significantly associated with self-efficacy of FSC and knowledge of DFU development.

Studies that investigated self-efficacy and effective FSC behaviors among DM patients revealed that DM patients with high-risk feet reported sufficient FSC practices [13], and self-efficacy was found to be a strong predictor for effective FSC behaviors among DM patients [14, 15].

Knowledge and awareness about appropriate FSC were found to be dependent on the level of education and the duration of DM [16]. In addition to the age of the patient, income level, marital status, and gender [17]. Poor educational level and low socio-economic status were found to be associated with lower awareness of appropriate FSC [2]. Finally, it was evident that older age DM patients were more confident in providing FSC and so had more self-efficacy in performing FSC [11, 18].

## 2 MATERIALS AND METHODS

### 2.1 Design and Sample

This was a secondary analysis of data collected from a cross-sectional multi-center study for all patients diagnosed with DM who had high-risk feet who were referred to King Abdullah University Hospital (KAUH) and the National Center for Diabetes, Endocrinology and Genetics (NCDEG) in the periods from October 2022 to March 2023. Inclusion criteria were Jordanian people of age > 18 years, able to give written informed consent and to communicate verbally, being diagnosed with type I or type II DM, and attending the diabetic foot clinics in either KAUH or NCDEG due to being high-risk feet patients. High-risk feet mean that a patient has a previous or current foot ulcer [19]. Exclusion criteria were illiterate patients with bilateral foot amputations, advanced diabetic retinopathy with severe impairment of vision, and any disease that might lead to cognitive impairment (*e.g.*, mental illness). The number of visits to diabetic foot clinics made by each participant should be more than 5 visits as repeated follow-up visits help in more adherence by DM patients and gaining more knowledge about their disease.

The minimum sample size needed by using G*power 3.1 [20] at α level of significance 0.05 (two-tailed), power of 0.8, and a medium effect size of 0.15 was 98 participants. The main outcome variable (*i.e.*: FSC) was continuous, and therefore multiple linear regression was employed to analyze the relationships of independent variables with FCS, with a calculation assuming 6 to 7 independent variables.

### 2.2 Ethical Approval and Data Collection

Ethical approval was obtained by the ethics committee at Al-Balqa Applied University (ethical approval number: 26/3/2/399). As our study involved human subjects, the Helsinki Declaration was followed by obtaining written consent from all participants included in our study. They acknowledged that their participation is voluntary, confidential, and implies answering a self-administered questionnaire. Data about patients’ demographics and clinical variables such as age, gender, marital status, level of education, duration of DM, and glycated hemoglobin levels (HbA1c) were obtained for each participant.

The questionnaire employed for data collection is the Arabic versions of the foot care outcomes expectations scale (FCOES), foot care confidence scale (FCCS), and patient interpretation of the neuropathy scale (PINS) [3]. FCOES includes 15 items that assess the patient’s outcome expectations of performing foot care and each item can be answered on a 5-point Likert scale [4]. PINS includes 11 items that estimate the knowledge of patients with DM of the possible causes of DFUs, with each item answered on a 5-point Likert scale [4]. The Arabic version of the FCOES was proved to be reliable and valid (content validity index =0.97, Cronbach’s alpha=0.97) [3]. The Arabic version of PINS has also been proven to be reliable (interclass correlation coefficient = 0.85) [3], and confirmed for criterion validity [4].

PINS items are divided into 3 subscales: self/ practitioner blame (blaming either self or care provider about developing neuropathy), physical causes of DFUs (physical problems that indicate or lead to developing neuropathy), and acute foot ulcer onset (timing of developing neuropathy) [4], and all of the subscales measure knowledge about DN [3]. The FCCS consists of 12 items answered on a 5-point Likert scale that measures confidence to perform FSC, which in turn helps healthcare providers in evaluating and enhancing outcomes of FSC in persons with DM [21]. In addition, FCCS is an indicator of self-efficacy for FSC activities [3]. The Arabic version of FCCS has an accepted internal consistency and was proven to be reliable (Cronbach’s alpha = 0.88) [3].

### 2.3 Statistical analysis

Analysis of study data was conducted *via* SPSS 22 [22]. Data was represented as frequencies and percentages (for categorical variables), as well as means (or medians) and standard deviations (SD) (for continuous variables). Multiple linear regression analyses were conducted to assess whether demographic and clinical variables are associated with foot care outcome expectations (FCOE), foot care confidence, and interpretation of neuropathy. A *p*-value less than 0.05 was considered significant.

## 3 RESULTS

### 3.1 Demographic and Clinical Characteristics of the Sample

Of the 150 patients who attended diabetic clinics in KAUH and NCDEG and met the inclusion criteria, 110 completed the questionnaire. Three questionnaires were not included in the data analysis due to a large amount of missing data: those participants were all older and had a lower level of education; therefore, the final sample included 107 patients.

The characteristics of the patients who participated in the study are summarized in Table **1**. Most patients were males, married, unemployed, non-smokers, and not living alone in cities. About two-thirds of them received higher education. Clinically, the majority of patients had type II diabetes, with poorly controlled DM as evidenced by high levels of HbA1c (*i.e.*, a value of HbA1c greater than 6% [23]). However, most patients were not receiving insulin treatment.

Regarding the chronic complications of DM, the majority of patients were not affected by heart failure, renal failure, or coronary artery disease (CAD). On the other hand, most patients reported retinopathy and hypertension. The protective sensation in the foot was mostly evident among patients. However, the majority of them had non-palpable foot pulses.

The average overall score of patients in FCCS was 47 ± 11.4 (SD) (60 is the highest score), in FCOES: 63 ± 10 (SD) (75 is the highest score), PINS: self/ practitioner blame: 12.8 ± 3.2 (SD) (20 is the highest score), PINS: physical causes of DFUs: 15.1 ± 3 (SD) (20 is the highest score), and PINS: acute ulcer onset: 10.8 ±2.1 (SD) (15 is the highest score) (Table **2**). In general, the scores on each of the above scales are moderate to high, which indicates good knowledge about DN, good FCOE, and moderately increased FSC confidence and, therefore, moderate to high level of self-efficacy for FSC. However, a difference between patients in the 2 research settings in the above scales was significant in the PINS: acute ulcer onset and PINS: self/ practitioner blame subscales (*p*<0.05) (Table **2**).

### 3.2 Factors Associated with Self-efficacy for FSC

The bivariate analysis revealed that FCCS was significantly associated with age, marital status, and having a heart attack or being diagnosed with CAD, so these three variables were included in the final regression analysis. Table **3** shows that the overall regression model was significant (adjusted *R^2^* = 0.046, *F* (3,103) = 2.72, *p* <0.05). However, age only was significantly associated with self-efficacy for FSC (*β* = -0.22, *p* <0.05); the other two variables were not significantly associated with self-efficacy for FSC among DM patients (*p* >0.05). Older ages were associated with a lower level of self-efficacy for FSC among DM patients.

### 3.3 Factors associated with FCOE

The bivariate analysis revealed that confidence in FSC and living arrangements were significantly associated with FCOE. Table **4** shows that the final model of multiple regression was significant (adjusted *R^2^* = 0.19, *F* (3,101) = 9.30, *p* <0.001). The most significant predictor in the regression model above is the FCCS (*β* = 0.39, *p* <0.001), which suggests strong relationships between confidence in FSC and FCOE. Patients who lived with family had higher FCOE compared to those who lived alone (*β* = 0.19, *p* <0.05).

### 3.4 Factors Associated with Interpretation of Neuropathy (PINS)

Bivariate analysis was conducted with independent variables for each subscale in PINS so that only significantly associated variables are included in the final regression analysis (Table **5**).

The final model of multiple linear regression for self/ practitioner blame subscale was significant (adjusted *R^2^* = 0.18, *F (*4,102) = 6.77, *p* <0.001). Self/ practitioner blame was significantly associated with three variables (*p* <0.05): diagnosis with renal failure (*β* = 0.20), having a heart attack or being diagnosed with CAD (*β* = -0.18), and research setting (*β* = 0.32). Renal failure and the settings in which the patient is receiving care for DM were associated with higher knowledge about the interpretation of neuropathy, and having a heart attack or being diagnosed with CAD was associated with lower knowledge about the interpretation of neuropathy.

Regarding the acute DFUs onset subscale, the final model of multiple linear regression was significant (adjusted *R^2^* = 0.09, *F* (4,101) = 3.70, *p* <0.05). Acute DFUs onset subscale was significantly associated with 2 variables (*p* < 0.05): research settings (*β* = 0.21) and HbAIC level (*β* = 0.25), which means that patients who received care in KAUH had higher knowledge of acute DFU onset more than patients who received care in NCDEG, and patients with higher levels of HbA1c were more knowledgeable about acute DFUs onset more than patients with lower levels of HbA1c.

The final multiple linear regression model for physical causes of DFUs subscale was also significant (adjusted *R^2^* = 0.10, *F* (3,103) = 5.05, *p* <0.05). Both the patient’s age ((*β* = -0.26) and diagnosis of retinopathy (*β* = 0.27) were significantly associated with the physical causes of DFUs subscale (*p* < 0.05). Older patients had a lower level of knowledge of the physical causes of DFUs than younger patients, and those diagnosed with retinopathy had higher knowledge of the physical causes of DFUs compared to patients who were not diagnosed with retinopathy. Level of education was not associated with knowledge of the physical causes of DFUs among DM patients (*p* >0.05).

## 4 DISCUSSION

To the best of our knowledge, this was the first study assessing the knowledge about FSC among patients with DM who have high-risk feet in Jordan as well as assessing FCOE in patients with DM, in addition to assessing how the demographic and clinical variables of DM patients were associated with FCOE, FSC self-efficacy, and knowledge about DFUs development.

We found that age was negatively associated with self-efficacy for FSC and as a result, there was less self-efficacy in undertaking FSC among older DM patients. Perrin *et al.* [18] reported that older patients have difficulty in performing basic behaviors of FSC (*i.e.*, foot inspection and nail trimming). Older adults were found to be less confident in performing basic FSC due to the presence of vision impairment and other medical problems that restrict their ability to perform basic FSC [11]. However, Sharoni *et al.* [24] found in their randomized trial study that older DM patients were more confident in practicing FSC behaviors and believed that FSC is a means of protection for their feet, and they usually learn from their personal experiences and share experiences with others. Also, Perrin *et al.* [25] found in their cross-sectional study on older DM patients over 50 years old that they were very confident about performing FSC. In our study, both young and older age patients with DM were included, which made our results different from the reported results above, and they included elderly patients in their study samples. From the discussion above, we can conclude that the relationship between age and confidence in FSC may vary across settings and contexts, thus confidence in providing FSC should be assessed among all age groups, and then support provided based on the level of self-efficacy for FSC. Enabling patients with DM to perform their own FSC and not relying on others is a major factor in enhancing FSC confidence [25], and conducting educational programs that can enhance self-efficacy for FSC may be beneficial for older DM patients to raise their confidence in performing FSC [24].

The results of our study have revealed that higher self-efficacy for FSC and having family support were associated with higher FCOE. This is consistent with the international literature. A previous study from Saudi Arabia found similar results in which self-efficacy to FSC was significantly associated with FCOE [26]. A cross-sectional study conducted in China [15] also found a strong association between confidence, self-efficacy, knowledge about FSC, and consequently effective FSC behaviors. Indeed, adherence to self-care behaviors is a result of strong beliefs in the valuable outcomes of self-care activities [10].

Social cognitive theory explains outcome expectations perception as a factor to be correlated with self-efficacy [27]. The correlation between self-efficacy and outcome expectations from FSC in the current study is interesting as health interventions to enhance any of these cognitive beliefs (*e.g.* programs to practice FSC skills, peer support) can be beneficial to improve either patients’ self-efficacy and positive outcome expectations about FSC. Future health education interventions to improve FSC might benefit from targeting these cognitive beliefs to enhance FSC.

Family members are integral in promoting FSC by providing assistance and advice and being a source of emotional support while managing DFUs, especially in tasks that patients with DFUs cannot perform [28]. Reisi *et al.* [10] also reported that family is considered an integral source of support that fosters the ability to manage disease in DM patients.

Our research also revealed that the self/ practitioner blame PINS subscale was associated positively with concurrent diagnosis with renal failure and the settings in which the patient is receiving care for DM. In our study, the two research settings (*i.e*: KAUH and NCDEG) have heterogeneous populations regarding living place (urban *vs.* rural), income, and education level; all of these might affect knowledge about DFUs and DN and, therefore, affect the level of this subscale results. This result was not surprising as similar findings regarding differences between the two research settings above were evident in a previous investigation [13] on factors related to FSC in DM patients. Educational and income level have been reported to be associated with DN [29], although in our study, they were not significantly associated with knowledge about neuropathy, and rural DM patients may have underdiagnosed DN that could lead to DFUs [30], which could be an important cause for blaming self or practitioner about having DN.

Being diagnosed with renal failure was associated with higher knowledge about the self/practitioner blame PINs subscale. One of the reasons that may explain this higher knowledge about DN and DFUs is the early detection of neuropathy in DM patients at early stages of decreased kidney function [31], and multiple visits to healthcare facilities for medical monitoring can expose DM patients to more health education [16], and so being knowledgeable about both renal impairment and neuropathy.

Having a heart attack or being diagnosed with CAD were associated with lower knowledge about the interpretation of neuropathy, in the self/practitioner blame subscale. Comorbidities in patients with DM, such as cardiovascular diseases (for example, hypertension, dyslipidemia, and CAD), increase the risk of having DN [5, 32, 33]. It is previously known that DN is strongly linked with asymptomatic heart attacks [34].

Furthermore, when a heart attack or CAD is associated with neuropathy, the DM patient will have silent ischemia and experience no pain sensation [35], which means that those patients are not aware or knowledgeable about DN and subsequent CAD or heart attacks. Moreover, it was reported that DM patients with DN had low knowledge about the causes and treatment of DN, and this low level of knowledge can be attributed to discordance among DM patients and healthcare providers regarding the impact of DN and how this topic is discussed during clinical visits [36]. As a result, DM patients should be equipped with sufficient knowledge about dealing with and preventing cardiovascular comorbidities as well as DN through appropriate health education from medical personnel in the healthcare institutions where they receive medical care.

The Acute DFUs onset PINS subscale was found to be positively associated with the settings in which patients are receiving care and elevated HbA1c levels. As mentioned above, the difference in both settings of our study might affect the knowledge level about DFUs and DN and the understanding of their nature, and this may be attributed to socioeconomic factors that differ between patients in both settings, such as income and educational level [13]. For this reason, greater emphasis should be placed on satisfied patient-provider communication as it is correlated with more understanding of DM as a chronic disease [28]. Furthermore, the medical team concerned with DFU treatment should establish comprehensive communication with patients at all stages of clinical treatment and acknowledge patients' concerns and worries about DFUs [37]. Chronic hyperglycemia (*i.e.*: higher and incontrollable blood glucose levels) in DM patients is one of the strong factors leading to the development of DFUs [38, 39], and thus those patients with elevated HbA1c levels are supposed to be knowledgeable about DFUs onset, as DM patients with medical complications understand their health condition better than those DM patients without medical complications [40]. Al Goblan *et al.* [41] found in their study in Saudi Arabia that elevated HbA1c levels are associated with poor prognosis of DFUs and delayed healing time of DFUs. Moreover, Akyuz *et al.* [42] revealed that elevated HbA1c levels were associated with DFUs onset and development of higher degrees of DFUs and, as a result, more risk for poor foot health and more tissue loss, and eventually more vulnerability for foot amputation. The majority of DM patients believe that controlling blood sugar levels helps control DN symptoms [36]. DM patients with a longer duration of DM will have higher HbA1c levels, and therefore, they will undergo multiple visits to healthcare institutions and be exposed to more education about their health status [16], which in turn contributes to having more knowledge about DN and acute DFUs onset.

Knowledge about the physical causes of DFUs /PINS subscale was found to be associated negatively with age and positively with a diagnosis of retinopathy. As mentioned above, DM patients with comorbidities such as retinopathy are more likely to develop DFUs than DM patients without comorbidities [5]. It is expected that DM patients with retinopathy undergo multiple visits to healthcare facilities, which means being more educated about their illness and, therefore, understanding their health condition better than those without retinopathy [40]. Therefore, retinopathy is considered a predictor of high adherence to FSC among DM patients, indicating better knowledge about DFUs [13]. Aging can increase the risk of DN due to the changes that occur in the neurological system [43], and older DM patients were found to be less knowledgeable and oriented to their health status and DM self-care, particularly DN and DFUs [44]. For the reasons above, older DM patients should receive more education and medical follow-up as they have less knowledge about DFUs and DN.

Our study has several limitations. Patients from the two research settings did not undergo exclusion criteria for several comorbidities that could help match patients from the two research settings, and the study was conducted with a cross-sectional design from two university hospitals only and therefore cause and effect could not be determined, which means that results about FSC generated by self-reports by study participants have to be treated cautiously. Further, some of the study variables such as the history of comorbidities are self-reported by the participants, making them prone to participants self-biases. Finally, most of the R square values are very low, indicating that there are other factors influencing the outcome variables that were not measured - needing further research to identify them.

## CONCLUSION

In conclusion, patients with DM in our study in two university centers in Jordan had a moderate to high level of self-efficacy to perform FSC, and older patients had less self-efficacy in foot care than younger patients. Patients who lived with family had higher FCOE compared to those who lived alone, and a strong relationship exists between confidence in FSC and FCOE. Differences existed between the two research settings regarding knowledge about DN and DFUs. Older patients with a history of heart attacks and CAD had less knowledge about DN and DFUs, and patients with high levels of HbA1c, diagnosed with retinopathy or renal failure were more knowledgeable about DN and DFUs than patients with lower levels of HbA1c and not diagnosed with retinopathy or renal failure. Future research should focus on the effect of continuous health education and the effect of self-efficacy programs in enhancing knowledge about DN and DFUs and consider self-efficacy in prevention programs by including the factors that improve self-efficacy, such as education, practical skills demonstrations, and peer support.

## Figures and Tables

**Table 1:** Participants demographic and clinical characteristics (mean (SD) or Number (%)) *N* = 107.

**Characteristic**	***n (%)* or mean (*SD*)**
**Demographic Characteristics**	
Research settings: NCDEG KAUH	50 (46.7) 57 (53.3)
Gender: Male Female	73 (68.2%) 34 (31.8%)
Age	58.52 (12.78)
Marital status: Single Married	28 (26.2%) 79 (73.8%)
Living arrangements: Living alone Living with family	9 (8.4%) 98 (91.6%)
Level of education: School education High education	74 (69.2%) 33 (30.8%)
Monthly family income	479.52 (311)
Living place: City Rural	83 (77.6%) 24 (22.4%)
Number of household members	4.92 (3.17)
Current employment status: Employed Unemployed	20 (18.7%) 87 (81.3%)
Medical insurance: Yes No	86 (80.4%) 21 (19.6%)
Smoking status: Non-smoker Current smoker	68 (63.6%) 39 (36.4%)
**Clinical Characteristics**	
Type of DM: Type I Type II	16 (15%) 91 (85%)
BMI:	29.52 (5.62)
Duration of DM	20.17 (11.45)
HbA1c	8.08 (1.85)
Treatment of DM: Insulin Non-insulin	33 (30.8%) 74 (69.2%)
Diagnosis with renal failure: Yes No	11 (10.3%) 96 (89.7%)
Diagnosis with heart failure: Yes No	24 (22.4%) 83 (77.6%)
Diagnosis with retinopathy: Yes No	75 (70.1%) 32 (29.9%)
Having a heart attack or being diagnosed with CAD: Yes No	40 (37.4%) 67 (62.6%)
Diagnosis with hypertension: Yes No	74 (69.2%) 33 (30.8%)
Presence of protective sensation: Present Absent	80 (74.8%) 27 (25.2%)
Foot pulses: Palpable Non- palpable	35 (32.7%) 72 (67.3%)
Deformities in the foot: Evident Not- evident	54 (50.5%) 53 (49.5%)

**Table 2:** Patients’ scores on FCCS, FCOES, and PINS (mean ± SD) (*N* = 107).

**Scale**	**Total**	**KAUH**	**NCDEG**	***p* Value**
FCCS	46.12 ± 9.66	45.37 ± 11.36	46.98 ± 7.28	0.39
FCOES	63.90 ± 9.47	64.67 ± 10.11	63 ± 8.67	0.37
PINS: self/ practitioner blame	14.21 ±4.14	15.46 ±3.24	12.78 ± 4.61	0.001*
PINS: physical causes of DFUs	15.73 ± 3.37	16.25 ±2.99	15.14 ± 3.71	0.09
PINS: acute ulcer onset	11.31 ± 2.29	11.72 ± 2.05	10.84 ± 2.48	0.047*

Note: * Significant at α = 0.05.

**Table 3:** Associations between variables and FCCS (*N* = 107).

**Variables**	**Unadjusted Association (*β*)**	**Adjusted Association (*β*)**	***p-value***
Age	-0.17	-0.22	0.03*
Marital status	2.07	0.1	0.33
Having a heart attack or being diagnosed with CAD	-0.88	0.04	0.66
*R* = 0.27 *R^2^*= 0. 073 *R^2adj^* = 0.046*			

Note: * statistically significant at α=0.05.

**Table 4:** Associations between variables and FCOES (*N* = 107).

**Variables**	**Unadjusted Association (*β*)**	**Adjusted Association (*β*)**	***p-value***
Living arrangements	6.31	0.19	0.04*
Foot pulses	2.60	0.13	0.15
FCCS	0.38	0.39	<0.001
*R* = 0.47 *R^2^*= 0.22 *R^2adj^* = 0.19**			

Note: * statistically significant at *α* = 0.05. ** statistically significant at *α* = 0.001.

**Table 5:** Associations between variables and PINS (*N* = 107).

**1. Associations between variables and self/practitioner blame**				
**Variables**	**Unadjusted Association (*β*)**	**Adjusted Association (*β*)**		***p-value***
Treatment of DM	1.479	.166		.063
Diagnosis with renal failure	2.660	.196		.032*
Having a heart attack or being diagnosed with CAD	-1.556	-.183		.045*
Research setting	2.605	.315		<.001**
*R* = 0.46 *R^2^*= 0.21 *R^2adj^* = 0.18**				
***2.* Associations between variables and physical causes of DFU**				
**Variables**	**Unadjusted Association (*β*)**		**Adjusted Association (*β*)**	***p-value***
Age	-.067		-.255	.007*
Level of education	1.049		.144	.121
Diagnosis with Retinopathy	1.949		.266	.005*
*R* = 0.36 *R^2^*= 0.13 *R^2adj^* = 0.10*				
**3. Associations between variables and acute DFU onset**				
**Variables**	**Unadjusted Association (*β*)**		**Adjusted Association (*β*)**	***p-value***
Living arrangements	-1.089		-.151	.108
HbA1c Level	.267		.245	.011*
Diagnosis with hypertension?	.750		.171	.076
Research Setting	.825		.205	.032*
*R* = 0.36 *R^2^*= 0.13 *R^2adj^* = 0.09*				

Note: * statistically significant at *α* = 0.05. ** statistically significant at *α* = 0.001.

## Data Availability

All data generated or analysed during this study are included in this published article.

## LIST OF ABBREVIATIONS

FCOE: Foot Care Outcomes Expectations
DM: Diabetes Mellitus
DFUs: Diabetic Foot Ulcers
DN: Diabetic Neuropathy

## STANDARDS OF REPORTING

STROBE guidelines were followed.
